# Supplementary material for: “Wounds Home Alone”—Why and How Venous Leg Ulcer Patients Self-Treat Their Ulcer: A Qualitative Content Study
Source: Int J Environ Res Public Health. 2019 Feb 15;16(4):559. doi: 10.3390/ijerph16040559 (PMC6406886; doi:10.3390/ijerph16040559)
Supplement: Supplementary file 1 [file ijerph-16-00559-s001.pdf]

Semi-structured questionnaire

1. How long do you have ulcer/ulcer? Is it the first time, or you had it before?
2. What do you think is the cause of ulcer?
3. Describe what medical treatment was done for ulcer healing?
4. Please describe the local wound treatment you had for your ulcer.
5. What is the local treatment in the last 30 days?
6. Do you apply compression therapy? What kind? Do you know what the purpose of compression therapy is?
7. Which treatment do you find the most efficient for ulcer healing?
8. Do you self treat your wound? When and why? Please describe what you do?
9. Did you sometimes think about changing local wound therapy by yourself? Please let me know why? What did you do?
10. Where you get information about your ulcer therapy?
11. Does someone help you with self treat? Who? How he/she helps you?
12. In overall, what do you find as the biggest obstacle/problem in healing your wound and generally in wound healing?

Codes for themes

In Table S1. presented are codes which arose from VLU patients interviews and made theme "Current local wound therapy." These codes present medications and devices recommended by clinicians to patients. Although the study is qualitative, number and percentages are displayed to give detailed insight. Some of these preparations are used alone and some in combination with others.

Table S1: Codes for theme "Current local wound therapy"

**Medication/device**	***n****	**%**
Ointments and creams	20	62,5%
Wound rinsing with saline	14	43,8%
Silver dressing	8	25,0%
Other dressing	8	25,0%
Octenisept[1]	6	18,8%
Aquacel[2]	6	18,8%
Wound showering	5	15,6%
hydrogels	4	12,5%
Beloderm[3]	2	6,3%
Granuflex[4]	2	6,3%
permanganate	2	6,3%
UV lamp	2	6,3%
BiaIodine	2	6,3%
gauze	2	6,3%
Biatain[5]	1	3,1%
hydrogen	1	3,1%
Vinegar cream + wild chestnut cream	1	3,1%

*n = number

[1]Octenisept= disinfectants solution; [2] Aquacel – registered hydrofiber dressing; [3] Beloderm – topical corticosteroid cream; [4] Granufelx – registered hydrocolloid dressing; [5]Biatain – registered foam dressing

In Table S2 presented are codes which formed theme "VLU self- treatment." In this table, we also present different codes, which patients applied alone or in combination. However, it can be seen that patients for self-treat use a simple solution like saline or gauze, or saline and some dressing they have from previous treatments.

Table S2. Codes for theme "VLU self- treatment"

**Medication**	***n****	**%**
wound rinsing with saline	7	23
gauze	6	20
dressing	4	13
wound showering	3	10
wild chestnut cream	2	6
hazel dormouse fat	1	3
eggshell	1	3
hydrogen	1	3
Octenisept[1]	1	3
pork fat	1	3
rinsing with disinfectant	1	3
silver dressing	1	3
sour weed	1	3

**n* = number; [1] Octenisept - disinfectants solution
